# An *S*-band multimode reflector antenna for a satellite constellation tracking system

**DOI:** 10.1038/s41598-023-44941-7

**Published:** 2023-10-18

**Authors:** Handong Wu, Yuhui Ren, Yingying Wang, Kai Zhang

**Affiliations:** 1 Xi’an HengDa Microwave Technology Development Co., Ltd., Xi’an, China; 2 School of Information Science and Technology, Northwest University, Xi’an, China

**Keywords:** Electrical and electronic engineering, Electronics, photonics and device physics

## Abstract

A multimode reflector antenna is a new concept proposed by our team in recent years, which does not correspond to the use of the traditional multimode feed but to innovation in the reflector design. This paper presents an *S*-band multimode reflector antenna based on multimode reflector theory. To achieve a flat-top beam shape, the main reflector of the antenna is divided into a middle region and an edge region. The height difference between them is approximately *λ*_0_/4, and then, the reflected waves in different areas partially cancel out in the direction of maximum radiation. The voltage standing wave ratio of the antenna is less than 1.5 from 2.8 to 3.4 GHz (19.4%), and the gain is more than 29.2 dB in the same frequency band. At the same time, a good flat-top beam is achieved in the range of ± 2°. The antenna can be used for satellite constellation tracking and other systems that require high-gain flat-top beams.

## Introduction

**Figure 1:**
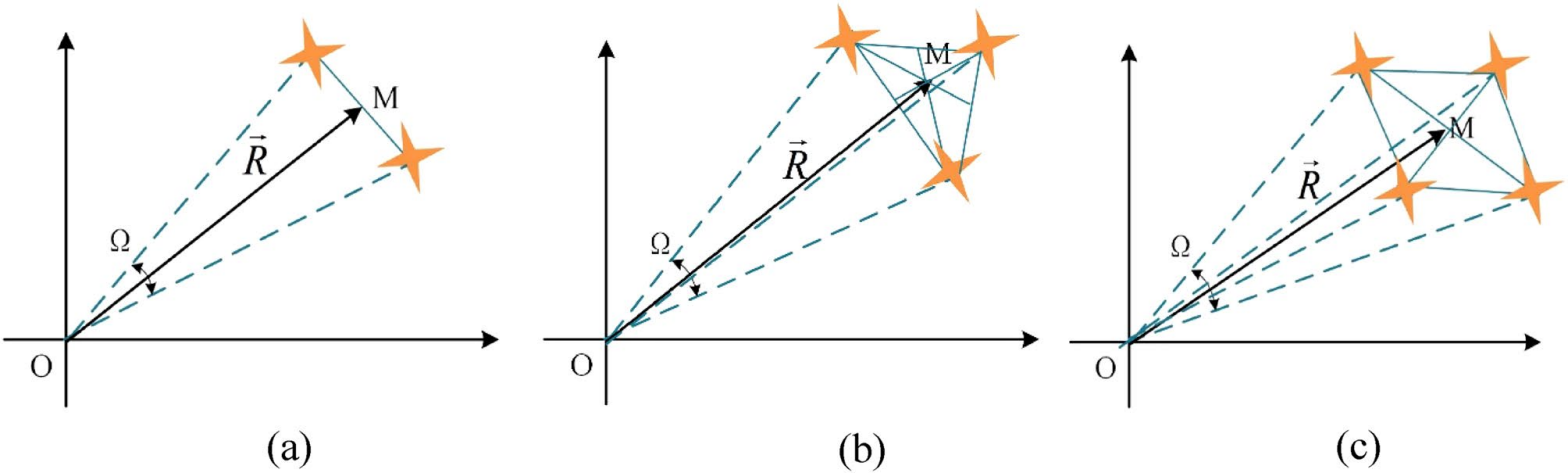
Satellite constellation diagram. (**a**) Shoulder pole constellation. (**b**) Triangular constellation. (**c**) Polygonal constellation.

In recent years, with the rapid increase in data traffic in navigation, remote sensing, communication and other satellite services, it has been difficult for a single satellite to meet the increasing application needs. Therefore, it has become a new trend of satellite services to use a multi-satellites “accompanying flight” network for data transmission. Especially in Nanosats and CubeSats networking, multiple small satellites can be used to form a constellation, as shown in Fig. 1, to realize the function of a large satellite^1–3^. For example, the characteristics of mobile communication satellite constellations in the mid-orbit zone are studied in^1^, and 10 constellation networking schemes are proposed, which can provide mobile communication services for more than 85% of people in the world. In reference to the atmospheric and oceanic data issued by the World Meteorological Organization, three remote sensing NanoSats satellite constellation schemes are designed in^2^, which can meet the minimum data requirements of numerical weather forecasting and other meteorological applications. In Ref.^3^, a low-Earth orbit satellite constellation scheme suitable for air traffic control is designed, which can realize global real-time air traffic monitoring.

**Figure 2:**
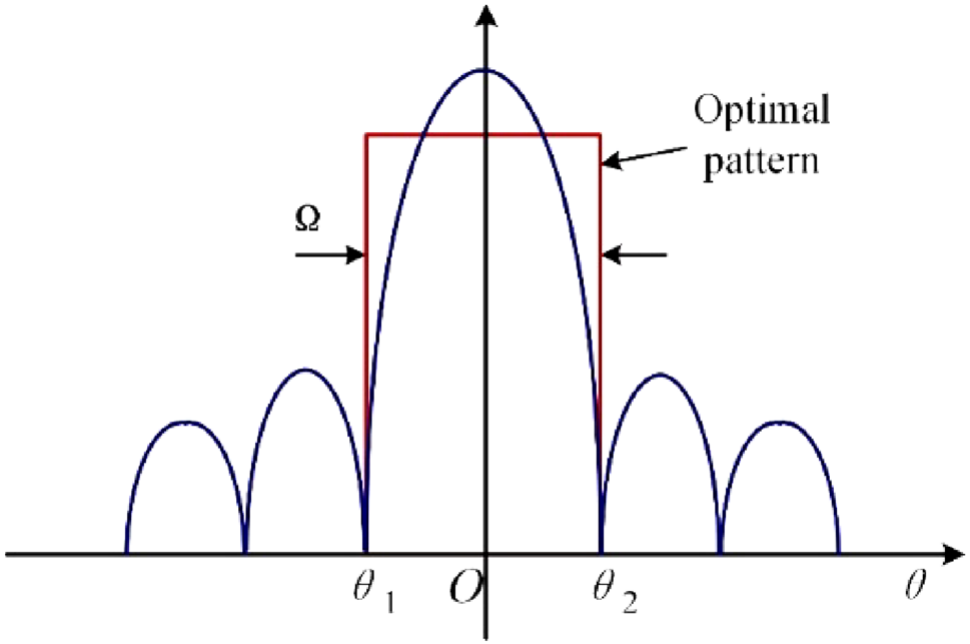
Constellation tracking optimal radiation pattern.

It can be seen from Fig. 1 that each constellation has a wide solid angle Ω to the earth, which is the visual range of the constellation. When we look at a constellation from the earth, the axis of vision generally points to the center of the constellation *M*, and each satellite is not in the maximum radiation direction ***R*** of the antenna. This results in wasted energy, and beam shift can cause the signal of each satellite to be unbalanced. Hence, to make the equipment on the ground have the same signal receiving and transmitting strength for each satellite, the beam of the antennas on the ground should have equal gain in the vision range of the constellation; that is, the optimal radiation pattern of the antennas should be a flat-top rectangular beam, as shown in Fig. 2. In addition, to capture and track satellite constellations, high-gain reflector antennas are often used in practical systems.

To design a reflector antenna with a flat-topped rectangular radiation pattern, the preferred solution is based on the theory of a shaped reflector; that is, the flat-topped pattern shown in Fig. 2 is taken as the objective function to design the mid-transversal of the reflector^4–6^. This method can feasibly achieve a wide-beam flat top pattern over a wide range of angles. In Ref.^6^, this method is used to design a biased reflector antenna operating at 19–22 GHz, which realizes a one-dimensional flat-top beam within ± 8° of the azimuth plane. In this range, the radiation level fluctuation is less than 0.6 dB, and the gain is more than 20 dB. However, the beam of high-gain antennas suitable for satellite systems is generally very narrow, and the gap between *θ*_1_ and *θ*_2_ in Fig. 2 is usually within 2°–4°. After many simulations, our team verified that it is difficult to achieve a high-gain and narrow-beamwidth flat-top pattern using traditional shaped reflector theory and that it is even more difficult to achieve a two-dimensional flat-top beam.

To solve the above problems, a new concept of a multimode reflector is proposed in this paper. Then, based on multimode reflector theory, two antiphase modes are generated in the maximum radiation direction of the biased reflector antenna so that a flat-topped or slightly concave pattern can be realized. The antenna has an impedance bandwidth of 19.4% from 2.8 to 3.4 GHz (VSWR| ≤ 1.5), a flat top pattern is achieved in the range of ± 2° in two dimensions, and the gain fluctuation is less than 1 dB. The antenna can be used for satellite constellation tracking and other systems that require high-gain flat-top beams.

## Multimode reflector antenna theory

**Figure 3:**
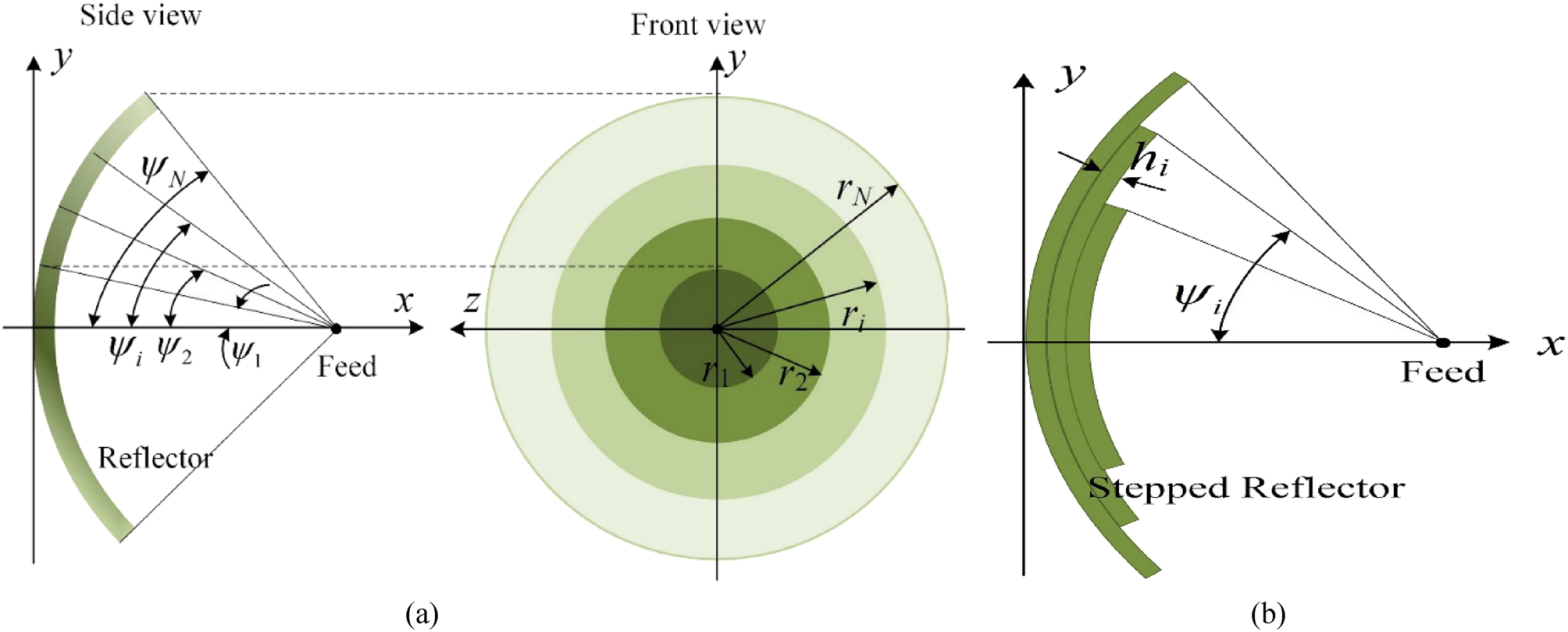
Multimode reflector antenna. (**a**) Fundamental form, (**b**) metal step-shaped reflector.

The basic structure of the multimode reflector is shown in Fig. 3a. The reflector is divided into a middle region and *N*-1 edge regions, and these regions are symmetrically distributed. The middle region excites the base mode (dominant mode), and the edge regions excite several different modes. By changing the amplitude and phase of the reflection coefficient Г_*i*_ in each region, multiple reflected waves with different modes are superimposed in the radiation direction, thus forming the desired high gain pattern with a special shape, where *i* = 1,2,…, *N*.

The general pattern function of a multimode reflector antenna can be expressed as:

1 $$ \begin{aligned} F(\theta ) & = \Gamma_{1} \int_{0}^{{\psi_{1} }} {\sqrt {G_{f} (\psi )} \tan \frac{\psi }{2} \cdot } J_{0} (u)d\psi + \Gamma_{2} \int_{{\psi_{1} }}^{{\psi_{2} }} {\sqrt {G_{f} (\psi )} \tan \frac{\psi }{2} \cdot } J_{0} (u)d\psi \\ & \quad + \cdots + \Gamma_{N} \int_{{\psi_{N - 1} }}^{{\psi_{N} }} {\sqrt {G_{f} (\psi )} \tan \frac{\psi }{2} \cdot } J_{0} (u)d\psi \\ & = |\Gamma_{1} |\int_{0}^{{\psi_{1} }} {\sqrt {G_{f} (\psi )} \tan \frac{\psi }{2} \cdot } J_{0} (u)d\psi + |\Gamma_{2} |e^{{2\alpha_{1} h_{1} }} e^{{ - j(4\pi h_{1} /\lambda_{g1} )}} \int_{{\psi_{1} }}^{{\psi_{2} }} {\sqrt {G_{f} (\psi )} \tan \frac{\psi }{2} \cdot } J_{0} (u)d\psi \\ & \quad + \cdots + |\Gamma_{N} |e^{{2\alpha_{N} h_{N} }} e^{{ - j(4\pi h_{N} /\lambda_{gN} )}} \int_{{\psi_{N - 1} }}^{{\psi_{N} }} {\sqrt {G_{f} (\psi )} \tan \frac{\psi }{2} \cdot } J_{0} (u)d\psi \\ \end{aligned} $$

where *G*_*f*_ (*ψ*) represents the pattern function of the feed, *α*_*i*_ and *λ*_*gi*_ represent the attenuation constant and dielectric wavelength of each region, respectively, and:

2 $$ u = \frac{kD}{2}\cot \frac{{\psi_{\max } }}{2}\tan \frac{\psi }{2}\sin \theta ,\quad J_{0} (u) = \frac{1}{2\pi }\int_{0}^{2\pi } {e^{j\mu \cos \xi } } d\xi $$

To achieve different Г_i_, the common methods are as follows: (1) each region is filled with a different dielectric material or resistive film; (2) metasurface structures with different characteristics are loaded in each area^7,8^; or (3) the metal reflector is processed into a stepped structure^9,10^. The antenna design in this paper adopts method (3) to realize a multimode reflector. As shown in Fig. 3b, the phase of reflected waves in different areas is controlled by the height of the step *h*_*i*_. Currently, Г_i_ = − 1, *α*_*i*_ = 0, and *λ*_*gi*_ = *λ*_0_, and Eq. (1) simplifies to (3):

3 $$ \begin{aligned} F(\theta ) & = - \int_{0}^{{\psi_{1} }} {\sqrt {G_{f} (\psi )} \tan \frac{\psi }{2} \cdot } J_{0} (u)d\psi - e^{{ - j(4\pi h_{1} /\lambda_{0} )}} \int_{{\psi_{1} }}^{{\psi_{2} }} {\sqrt {G_{f} (\psi )} \tan \frac{\psi }{2} \cdot } J_{0} (u)d\psi \\ & \quad - \cdots - e^{{ - j(4\pi h_{N} /\lambda_{0} )}} \int_{{\psi_{{N{ - }1}} }}^{{\psi_{N} }} {\sqrt {G_{f} (\psi )} \tan \frac{\psi }{2} \cdot } J_{0} (u)d\psi \\ \end{aligned} $$

The detailed derivation of Eqs. (1)–(3) is given in Appendix. It should be emphasized that the multimode reflector antenna is a new concept proposed by our team. First, the term “multimode” does not correspond to what is commonly referred to as a multimode primary feed but rather to an innovation in the structure of the reflector^11^. Second, reflected waves with different phase states are called multiple modes, which is a supplement to and an extension of the concept of modes defined according to the field distribution in bounded space, such as waveguides and horns. Third, multimode reflectors are different from stepped reflectors. A stepped reflector is only one way to realize a multimode reflector, which happens to be used in this paper.

## Antenna design and analysis

**Figure 4:**
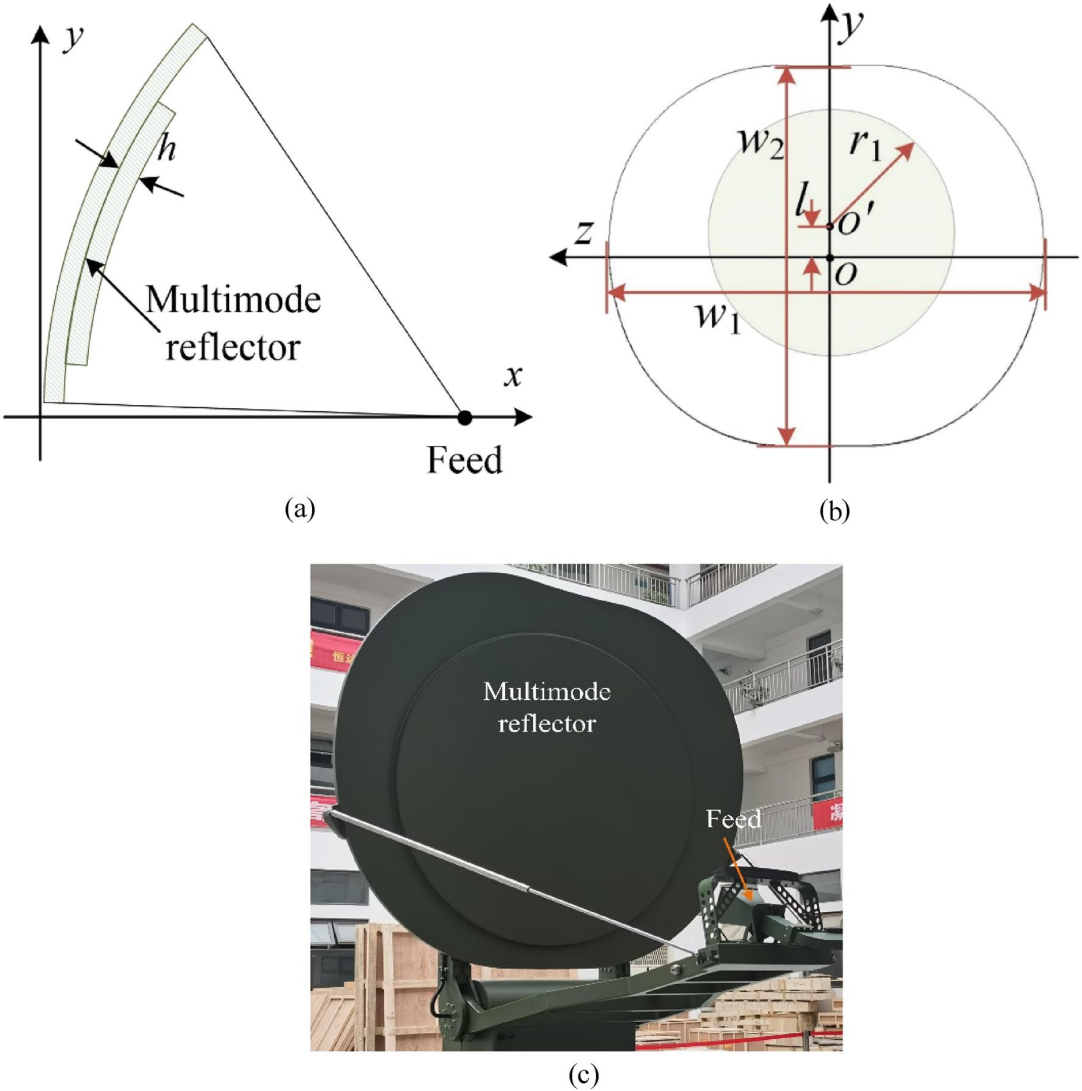
Antenna structure: (**a**) side view; (**b**) front view; (**c**) physical photograph.

The basic structure of the multimode reflector antenna proposed in this paper is shown in Fig. 4, which is mainly composed of a horn feed and a multimode reflector. When in use, the antenna is installed on the top of a vehicle cabin, so the form of a partial feed is adopted for easy folding and storage. Due to the limited space of the roof, the main body of the reflector is cut from an elliptical reflector. To obtain two antiphase patterns, the main reflector is divided into a middle region and an edge region. The height difference between the two regions is *h*; that is, the reflected wave in the edge region can produce a phase lag compared with the middle region:

4 $$ \Delta \varphi = 2kh = 4\pi h/\lambda_{0} $$

where *k* represents the phase constant in free space. When *h* = *λ*_0_/4, the phase difference of the aperture fields reflected in the two regions is 180°, and two antiphase modes can be formed. Considering the gain, size, weight and other factors, the final size of the edge region *w*_1_ × *w*_2_ ≈ 24*λ*_0_ × 20*λ*_0_, the focal diameter ratio of the antenna is 0.68, and the irradiation angle of the feed is 63.5°.

When *w*_1_ and *w*_2_ are determined, the ratio of the two modes (0 and π) can be adjusted by adjusting the radius *r*_1_ of the middle circular area. In addition, as seen in Fig. 4b, the center of the edge area* o* does not coincide with the center of the middle region* o*′, which can solve the problem of asymmetry of the radiation pattern in the *y* direction caused by irregular cutting of the edge area.

**Figure 5:**
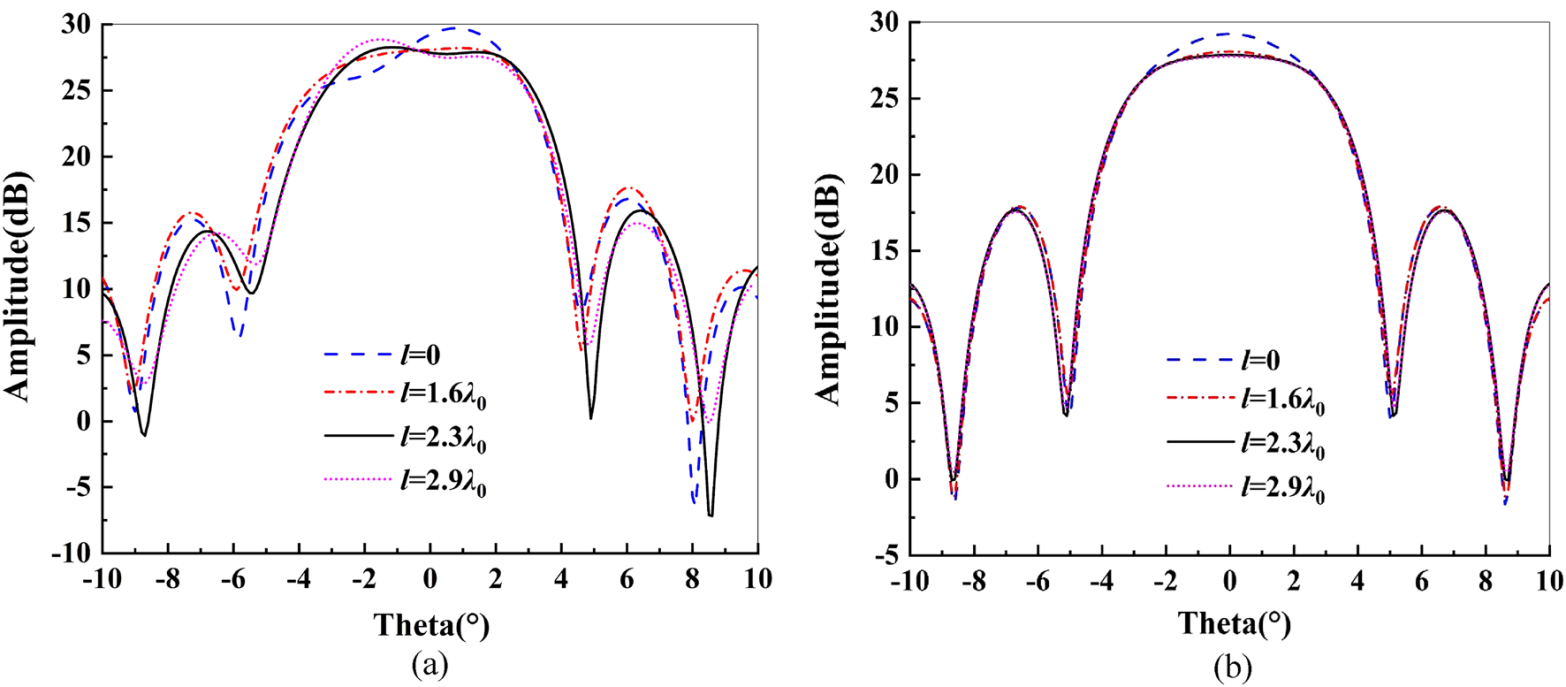
Influence of parameter *l* on the pattern: (**a**) E-plane; (**b**) H-plane.

As mentioned above, *o* and *o*′ do not coincide, and the offset *l* between them affects the performance of the reflector. We use full wave analysis software CST Studio Suite to simulate the performance of the antenna. Figure 5 shows the influence of different *l* values on the pattern of the antenna. When *l* = 0, *o* and *o*′ coincide. Since the edge area of the reflector is asymmetric along the *y* direction, the E-plane pattern of the antenna is also asymmetric. As *l* increases, the maximum gain of the antenna slightly decreases, and the pattern tends to be flat. However, if *l* continues to increase, then the E-plane pattern will tilt in the opposite direction. In addition, the symmetry of the H-plane pattern is not affected by the change in *l.* In this design,* l* = 2.3*λ*_0_ is finally determined.

**Figure 6:**
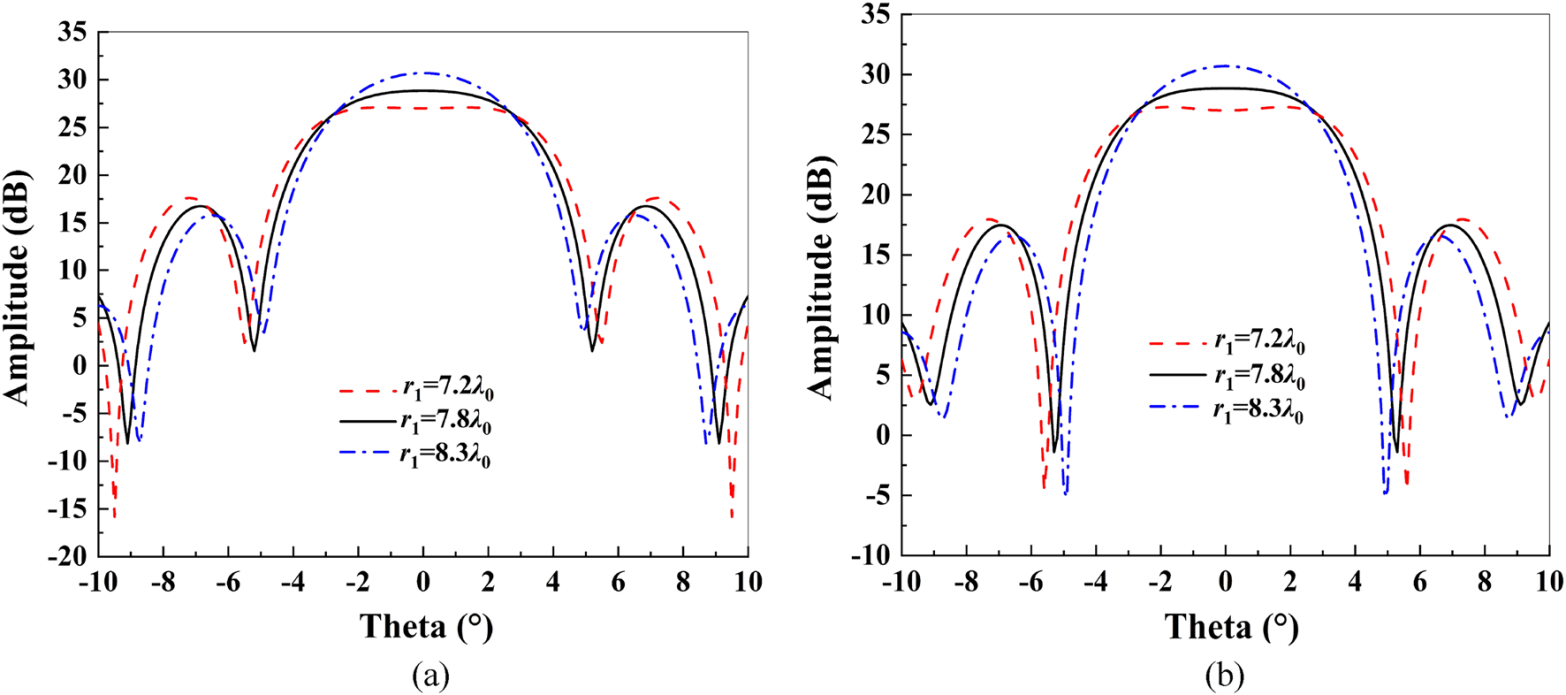
Influence of parameter *r*_1_ on the pattern: (**a**) E-plane; (**b**) H-plane.

Figure 6 shows the influence of parameter *r*_1_ on the radiation pattern of the antenna. When *r*_1_ increases, the gain will also increase, and the sidelobe level will gradually decrease. However, the beam width of the flat top area will become increasingly narrower. Therefore, comprehensively considering the beam width, maximum gain and sidelobe level, *r*_1_ = 7.8*λ*_0_ is finally determined.

**Figure 7:**
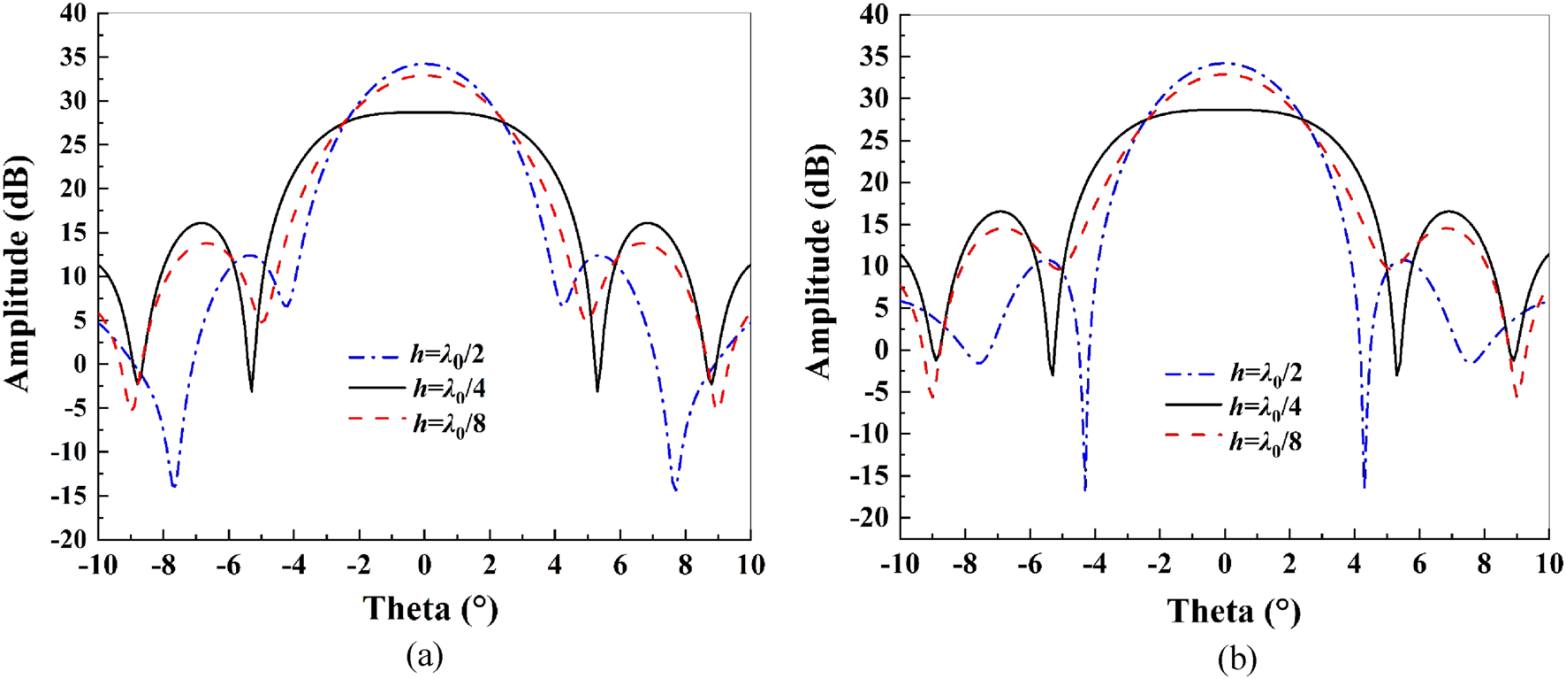
Influence of parameter *h* on the pattern: (**a**) E-plane; (**b**) H-plane.

The waves reflected in the two regions have different phase differences for different values of the height difference *h* between the middle region and the edge region of the reflector. For example, when *h* is *λ*_0_/8, *λ*_0_/4, and *λ*_0_/2, the corresponding phase differences are π/2, π, and 2π, respectively. In Fig. 7, the effect of different values of *h* on the antenna pattern is simulated, and it can be seen that when *h* = *λ*_0_/2, the reflected waves in different regions are superimposed in phase to produce the maximum gain. When *h* = *λ*_0_/4, the reflected waves cancel out in the direction of maximum radiation and then generate the desired flat-top beam. In addition, *h* also has an obvious influence on the sidelobe level of the antenna, which needs to be comprehensively considered.

**Figure 8:**
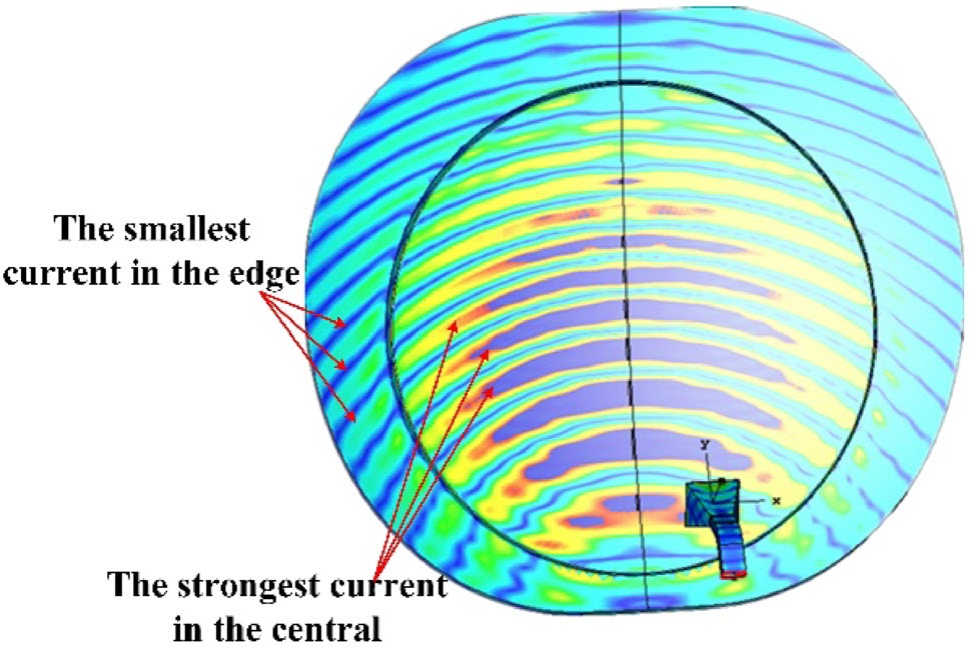
Multimode reflector surface current distribution.

Figure 8 shows the current distribution on the multimode reflector antenna. Because the edge region is shifted by *λ*_0_/4 compared to the middle region, the phases of their surface currents differ by π/2. When observed at the same latitude, the maximum value of the current in the middle region corresponds to the minimum value of the current in the edge region, and they are orthogonal.

## Results

**Figure 9:**
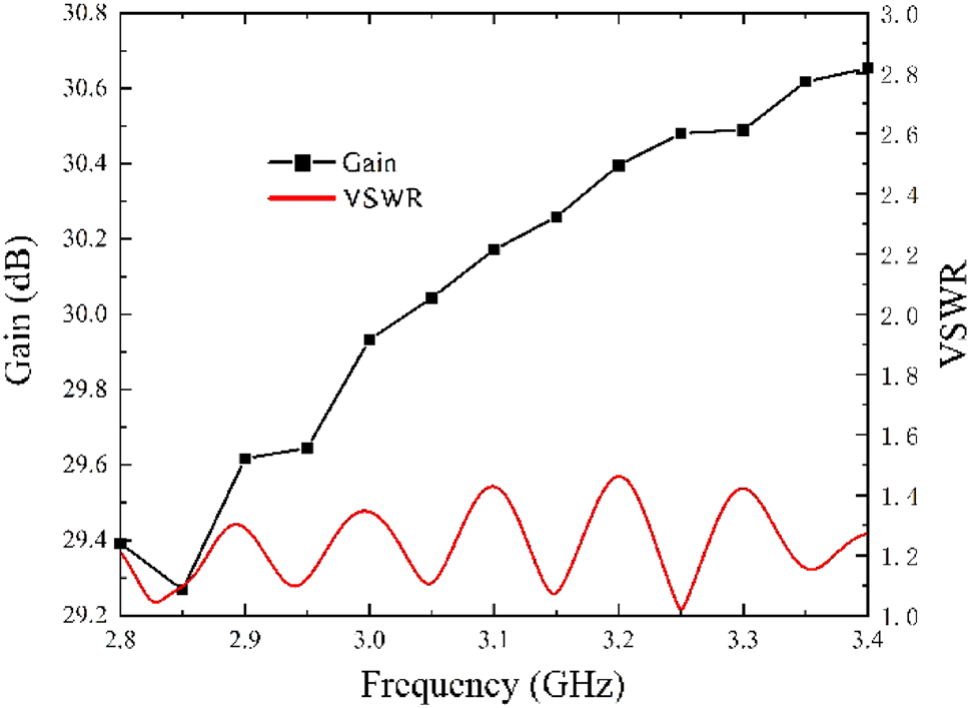
Measured gain and VSWR of the antenna.

The size of the antenna is determined and the actual antenna is processed according to the previous simulation analysis, as shown in Fig. 4c. Figure 9 shows the voltage standing wave ratio (VSWR) and gain of the antenna as a function of frequency, which shows that the VSWR of the antenna is less than 1.5 in the frequency range of 19.4% in the *S*-band. In the same band, the gain of the antenna is greater than 29.2 dB and up to 30.65 dB.

**Figure 10:**
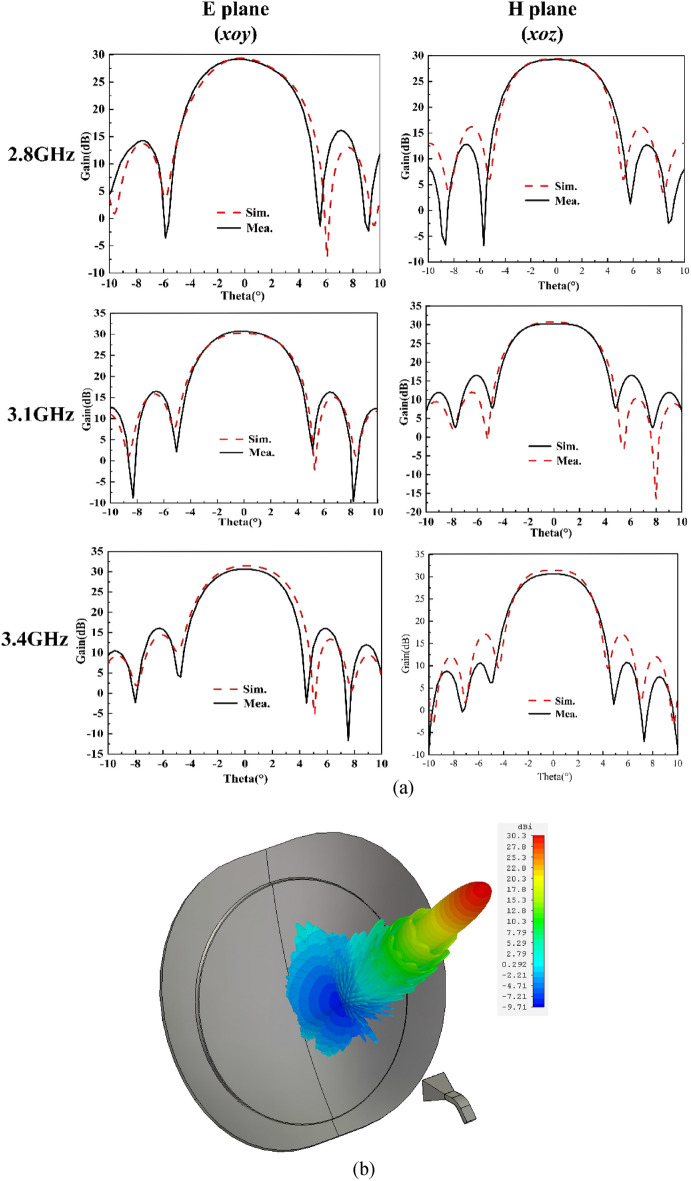
Measured and simulated radiation patterns of the antenna: (**a**) the pattern on the diagonal section; (**b**) simulated 3D radiation pattern at f = 3.1 GHz.

In Fig. 10a, the simulated and measured radiation patterns of the antenna at three frequency points are compared. It can be seen that the patterns have good symmetry in the *xoz* plane because the structure of the multimode reflector is symmetrical from left to right (Fig. 3b). However, in the *xoy* plane, the structural asymmetry causes the patterns to be skewed. Especially at 2.8 GHz, this phenomenon is more obvious, and the parameter* l* needs to be adjusted to achieve balance. In conclusion, the gain decrease of the presented antenna is less than 1 dB in the range of ± 2°, which indicates that it has good flat-top characteristics. The sidelobe level of the antenna is less than − 15 dB, which fully meets the application requirements of the satellite tracking system. In addition, Fig. 10b, shows the simulated 3D radiation pattern at *f* = 3.1 GHz, so that we can more intuitively observe the high-gain flat-top characteristics of the proposed antenna.

## Discussion

Multimode reflector theory is an innovation and an extension of the reflector antenna theory developed by our team in recent years, which can be used to design high-gain shaped-beam antennas. In this paper, an *S*-band multimode reflector antenna is designed, which can be effectively used in satellite constellation tracking systems. To achieve a flat-top radiation pattern, the gain of the antenna must be greater than 29.2 dB, and the maximum aperture size of the reflector reaches 24*λ*_0_. If only the thickness difference and areas of the middle and edge regions are adjusted, then disappointingly, the desired flat-top beam cannot be achieved. Therefore, the structure of the antenna is innovative; that is, the centers of the inner and outer regions of the reflector surface do not coincide. Finally, the VSWR of the antenna is less than 1.5, and the gain is more than 29.2 dB in the bandwidth of 19.4%. At the same time, a good flat-top beam is achieved in the range of ± 2°. It should be noted that the radiation efficiency of the traditional parabolic antenna is about 50%, while the efficiency of the proposed multimode reflector antenna is only 26%. This is because multimode reflector sacrifices the efficiency and maximum gain of the antenna to achieve a flat-topped beam.

### Supplementary Information

Supplementary Information.

## Data Availability

All data generated or analysed during this study are included in this published article.
