# Supplementary material for: An S-band multimode reflector antenna for a satellite constellation tracking system
Source: Sci Rep. 2023 Oct 18;13:17721. doi: 10.1038/s41598-023-44941-7 (PMC10584939; doi:10.1038/s41598-023-44941-7)
Supplement: Supplementary file 1 — Supplementary Information. [file 41598_2023_44941_MOESM1_ESM.docx]

Appendix

Fig. A. The geometry of a parabolic antenna.

It is well known that the radiation field of a parabolic antenna is the superposition of the aperture field and the leakage radiation of the feed. As illustrated in Fig. A, let the pattern of the feed be *G_f_* (*ψ*, ξ), then the electric field intensity at *M* on the aperture is expressed as:

$E_{M}(r,\xi)=\frac{\sqrt{60P_{\Sigma}G_{f}(\psi,\xi)}}{\rho}$ (a)

where *P_Σ_* represents the radiated power of the feed.

The radiation fields *E_E_* and *E_H_* in the far region can be calculated from the aperture field.

$\begin{matrix} \begin{matrix} E_{E}=\frac{jf\sqrt{60P_{\Sigma}}(1+\cos\theta)e^{-jkR}}{\lambda R}\int_{0}^{2\pi} \int_{0}^{\psi} \sqrt{G_{f}(\psi,\xi)}\tan\frac{\psi}{2}e^{j2kf\tan\frac{\psi}{2}\cos\xi\sin\theta}d\psi d\xi\text{,} & \mathrm{E}-\mathrm{plane} \end{matrix} \\ \begin{matrix} E_{H}=\frac{jf\sqrt{60P_{\Sigma}}(1+\cos\theta)e^{-jkR}}{\lambda R}\int_{0}^{2\pi} \int_{0}^{\psi} \sqrt{G_{f}(\psi,\xi)}\tan\frac{\psi}{2}e^{j2kf\tan\frac{\psi}{2}\sin\xi\sin\theta}d\psi d\xi, & \mathrm{H}-\mathrm{plane} \end{matrix} \end{matrix}$ (b)

Then the normalized pattern of antenna is obtained.

$\begin{matrix} \begin{matrix} F_{E}=\int_{0}^{2\pi} \int_{0}^{\psi} \sqrt{G_{f}(\psi,\xi)}\tan\frac{\psi}{2}e^{j2kf\tan\frac{\psi}{2}\cdot\cos\xi\cdot\sin\theta}d\psi d\xi, & \mathrm{E}-\mathrm{plane} \end{matrix} \\ \begin{matrix} F_{H}=\int_{0}^{2\pi} \int_{0}^{\psi} \sqrt{G_{f}(\psi,\xi)}\tan\frac{\psi}{2}e^{j2kf\tan\frac{\psi}{2}\cdot\sin\xi\cdot\sin\theta}d\psi d\xi, & \mathrm{H}-\mathrm{plane} \end{matrix} \end{matrix}$ (c)

The focal length to diameter ratio (*f /D*) of a parabolic antenna is expressed as

$\frac{f}{D}=\frac{1}{4}\cot\frac{\psi_{\max}}{2}$ (d)

Then

$\begin{matrix} \begin{matrix} F_{E}=\int_{0}^{2\pi} \int_{0}^{\psi} \sqrt{G_{f}(\psi,\xi)}\tan\frac{\psi}{2}e^{j\frac{kD}{2}\cot\frac{\psi_{\max}}{2}\tan\frac{\psi}{2}\cos\xi\sin\theta}d\psi d\xi, & \mathrm{E}-\mathrm{plane} \end{matrix} \\ \begin{matrix} F_{H}=\int_{0}^{2\pi} \int_{0}^{\psi} \sqrt{G_{f}(\psi,\xi)}\tan\frac{\psi}{2}e^{j2\frac{kD}{2}\cot\frac{\psi_{\max}}{2}\tan\frac{\psi}{2}\sin\xi\sin\theta}d\psi d\xi, & \mathrm{H}-\mathrm{plane} \end{matrix} \end{matrix}$ (e)

Because the feed pattern is symmetric, so

$G_{f}(\psi,\xi)=G_{f}(\psi)$ (f)

$F=F_{E}=F_{H}=\int_{0}^{\psi} \sqrt{G_{f}(\psi)}\tan\frac{\psi}{2}[\int_{0}^{2\pi} e^{j\mu\cos\xi}d\xi]d\psi$ (g)

where $u=\frac{kD}{2}\cot\frac{\psi_{\max}}{2}\tan\frac{\psi}{2}\sin\theta$.

Let

$$J_{0}(u)=\frac{1}{2\pi}\int_{0}^{2\pi} e^{j\mu\cos\xi}d\xi$$

Then

$F(\theta)=\int_{0}^{\psi} \sqrt{G_{f}(\psi)}\tan\frac{\psi}{2}\cdot J_{0}(u\text{）}d\psi$ (h)

The equation (h) illustrates the pattern of the classic parabolic antenna. For a multimode reflector antenna, the reflector is divided into an intermediate region and *N*-1 edge region, and each region has a different reflection coefficient Г*_i_*. Therefore, the pattern of the multimode reflector needs to be integrated piecewise over different regions. The equation (h) is rewritten as:

$\begin{matrix} F(\theta)=\Gamma_{1}\int_{0}^{\psi_{1}} \sqrt{G_{f}(\psi)}\tan\frac{\psi}{2}\cdot J_{0}(u\text{）}d\psi+\Gamma_{2}\int_{\psi_{1}}^{\psi_{2}} \sqrt{G_{f}(\psi)}\tan\frac{\psi}{2}\cdot J_{0}(u\text{）}d\psi\\ \begin{matrix} & & \end{matrix}+\cdot\cdot\cdot\cdot+\Gamma_{N}\int_{\psi_{N\text{-}1}}^{\psi_{N}} \sqrt{G_{f}(\psi)}\tan\frac{\psi}{2}\cdot J_{0}(u\text{）}d\psi\\ \begin{matrix} & \end{matrix}=|\Gamma_{1}|\int_{0}^{\psi_{1}} \sqrt{G_{f}(\psi)}\tan\frac{\psi}{2}\cdot J_{0}(u\text{）}d\psi+|\Gamma_{2}|e^{2\alpha_{1}h_{1}}e^{-j(4\pi h_{1}/\lambda_{g1})}\int_{\psi_{1}}^{\psi_{2}} \sqrt{G_{f}(\psi)}\tan\frac{\psi}{2}\cdot J_{0}(u\text{）}d\psi\\ \begin{matrix} & & \end{matrix}+\cdot\cdot\cdot\cdot+|\Gamma_{N}|e^{2\alpha_{N}h_{N}}e^{-j(4\pi h_{N}/\lambda_{gN})}\int_{\psi_{N\text{-}1}}^{\psi_{N}} \sqrt{G_{f}(\psi)}\tan\frac{\psi}{2}\cdot J_{0}(u\text{）}d\psi\end{matrix}$ (1)

which is the equation (1) in this paper. Where $\Gamma\text{=}|\Gamma|e^{2\alpha h}e^{-j(4\pi h/\lambda_{g})}$takes into account that different regions of the reflector have different attenuation constants and phase constants.

For the metal step - shaped multimode reflector, the Г_i_ =-1, *α_i_*=0, *λ_gi_*=*λ*_0_, and equation (1) simplifies to (3):

$\begin{matrix} F(\theta)=-\int_{0}^{\psi_{1}} \sqrt{G_{f}(\psi)}\tan\frac{\psi}{2}\cdot J_{0}(u\text{）}d\psi-e^{-j(4\pi h_{1}/\lambda_{0})}\int_{\psi_{1}}^{\psi_{2}} \sqrt{G_{f}(\psi)}\tan\frac{\psi}{2}\cdot J_{0}(u\text{）}d\psi\\ \begin{matrix} & & \end{matrix}-\cdot\cdot\cdot\cdot-e^{-j(4\pi h_{N}/\lambda_{0})}\int_{\psi_{N\text{-}1}}^{\psi_{N}} \sqrt{G_{f}(\psi)}\tan\frac{\psi}{2}\cdot J_{0}(u\text{）}d\psi\end{matrix}$ (3)
